# Birth weight influences differently on systolic and diastolic blood pressure in children and adolescents aged 8–15

**DOI:** 10.1186/s12887-022-03346-7

**Published:** 2022-05-13

**Authors:** Rui Huang, Shengxiang Yang, Yuhua Lei

**Affiliations:** Cardiovascular Disease Center, Central Hospital of Enshi Tujia and Miao Autonomous Prefecture, Enshi Clinical College of Wuhan University, No.158 Wuyang Avenue, Hubei Province 445000 Enshi City, China

**Keywords:** Birth weight, Blood pressure, Hypertension, NHANES

## Abstract

**Aim:**

Globally, hypertension is one of the main threats to public health and a significant risk factor predisposing individuals to various cardiovascular conditions. Hypertension in the young is particularly complex and challenging. Accumulating evidence has implicated that low birth weight is vital for elevated blood pressure, and birth weight was negatively correlated with blood pressure. However, fewer studies with conflicting results have addressed the associations between birth weight and blood pressure in children and adolescents, and there is no relevant research conducted in the NHANES population. The principal objective of this project was to investigate the relationship between birth weight and blood pressure in children and adolescents in NHANES.

**Methods:**

A total of 7600 subjects aged 8 to15 were enrolled in the present study from the National Health and Nutrition Examination Survey (NHANES) 2007–2018. Outcome variables were systolic blood pressure(SBP) and diastolic blood pressure(DBP). Birth weight was regarded as an independent variable. EmpowerStats software and R (version 3.4.3) were performed to examine the association between birth weight and SBP or DBP.

**Results:**

Birth weight was negatively correlated with SBP in the fully-adjusted model(β = -0.02, 95%CI: -0.04 to -0.04, *p* = 0.0013), especially in non-Hispanic White (β = -0.03, 95%CI: -0.06 to -0.00,*p* = 0.0446), aged between 13 to 15(β = -0.03, 95%CI: -0.04 to -0.01, *p* = 0.0027), and male individuals(β = -0.03, 95%CI: -0.05 to -0.01, *p* = 0.0027). However, there was no unidirectional association between birth weight and DBP in the fully adjusted model(β = -0.01, 95%CI: -0.03 to 0.02, *p* = 0.5668) and in sub-analysis. An inverted U-shaped and J-shaped relationship was uncovered between birth weight and DBP in those aged 13 or above and Mexican Americans, respectively. The inflection point calculated by a recursive algorithm of birth weight in these groups was all 105 oz.

**Conclusions:**

The current study identified that birth weight was negatively related to SBP but not significantly related to DBP in children and adolescents aged 8 to 15, highlighting different potential mechanisms behind high SBP and high DBP in the young. However, an inverted U-shaped and J-shaped relationship between birth weight and DBP was observed, suggesting that targeted intervention measures should be taken for different groups of people rather than generalizations.

## Introduction

Hypertension, a global threat to human health, substantially increases the risk of cardiovascular diseases and is a prominent contributor to mortality with more than 1 billion cases [1, 2]. Along with the improvement in the social economy and the aging of the population, the incidence of hypertension is rising at an alarming rate. In China, the prevalence of hypertension is 23.2% [3], while in the United States, the situation may be even more critical [4]. Notably, recent studies have also shown that high BP in adolescents and children is also a worrisome concern [5–7]. The United States is burdened with an increased incidence of hypertension in children and adolescents constantly, seriously hindering the development and health of the young [6, 8, 9].

Hypertension is a multifactorial disorder with complex mechanisms and numerous causes arising from the interplay of lifestyles, living conditions, and genetic factors [10, 11]. In addition, accumulating evidence has implicated that birth weight is a vital element for elevated blood pressure, implying that adverse effects in early life may be another etiology of hypertension. Various observational studies have detected an inverse relationship between birth weight and blood pressure [12–14]. For instance, Al Salmi I et al. [12] revealed in their study that birth weight was reversely associated with both SBP and DBP in a representative Austrian population. Consistent with the previous findings, a cross-sectional study in China suggested that low birth weight was related to an increased hypertension risk in adolescent girls, irrespective of BMI [13].

However, the current research on the relationship between birth weight and blood pressure is partly controversial and even completely contradictory [15–17]. Lai C et al. [16] found a U-shaped relationship between birth weight and blood pressure, especially SBP. They believed that birth weight and blood pressure were unidirectional but a complex relationship that is affected by a combination of various factors. Moreover, a sizeable Mendelian randomization analysis failed to detect an inverse relationship between birth weight and blood pressure [15]. Another similar study on birth weight and blood pressure in rural subjects also highlighted this issue [17].

Additionally, fewer studies with conflicting results have addressed the associations between birth weight and blood pressure in children and adolescents, and there is no relevant research conducted in the NHANES population. Therefore, we proposed this study to investigate the relationship between birth weight and blood pressure in NHANES, which has tremendous clinical implications for promoting the health of adolescents and reducing the risk of cardiovascular disease in adulthood.

## Methods

### Study population

The National Health and Nutrition Examination Survey (NHANES), a population-based survey conducted by the National Center for Health Statistics (NCHS), is a publicly used data set used to record the health status and related personal and lifestyle characteristics of all civilians in the United States. A multi-stage, complex clusters, probabilistic sample design is used for data acquisition and analysis to achieve a nationally representative rather than a simple random sample from the general US population [18]. In particular, the Centers for Disease Control and Prevention (CDC) is responsible for preparing and disseminating data files to provide full access to the data [19]. The present study analyzed the NHANES data collected from 2007 to 2018, which contained cross-sectional socio-demographic, dietary, and medical records obtained by questionnaires, standard physical examinations, and laboratory tests conducted in authoritative laboratories.

A total of 9094 subjects aged between 8–15 were enrolled in the primary analysis. The inclusion criteria were subjects who had self-reported birthweight with valid blood pressure records. We adhered to the following exclusion criteria:(1) individuals with missing birth weight or with invalid information; (2) those without blood pressure records;(3) those with self-reported severe diseases such as cancer and any other congenital diseases. The final sample size for analysis was 7600, after excluding the participants as mentioned above. The NCHS Ethics Review Committee supports this study, and each subject also signed a written informed consent [20].

### Variables

Blood pressure, including SBP and DBP, and birth weight were treated as dependent and independent variables, respectively. Proxy-reported birth weight data are only available for individuals aged 15 and under. Birth weight was recorded in pounds and ounces and converted into ounces in the final analysis. According to the World Health Organization (WHO) definition, low birth weight means that the birth weight is less than 2,500 g [21], which is about 88.2 oz. Therefore, we divide the research objects into two groups(< 88.2 oz and >  = 88.2 oz).

In addition, the following variables were collected and analyzed in the current study: age, race/ethnicity, gender, the ratio of family income to poverty, height, weight, body mass index (BMI), waist circumference, triglyceride (TG), total cholesterol (TC), LDL-cholesterol (LDL), plasma fasting glucose (FBG), uric acid (UA), creatinine (Cr), blood urea nitrogen (BUN) and glycohemoglobin. Also, associated risk factors, including the mother's age when the child was born, were also collected and analyzed. As BMI in the young varies with gender and age, we calculate age- and sex-specific BMI to evaluate weight status by using height and weight measurements gathered from the physical examination [22]. We defined overweight and obesity by using CDC criteria, which define overweight as age- and sex-specific BMI ≥ 85th percentile and obesity as BMI ≥ 95th percentile [23].

Blood pressure measurement procedure: After all subjects rested quietly for at least 5 min in the Mobile Inspection Center (MEC), the appropriate cuff was selected according to the size of the subject's upper arm. Three consecutive blood pressure readings will be obtained once the participant's maximum inflation level (MIL) is determined. If the measurement is interrupted or incomplete, or invalid, a fourth reading will be undertaken. An average value of all valid readings was served as the final blood pressure.

Missing value management: We excluded subjects with missing independent or dependent variables. The missing continuous variables were replaced by the median. The missing categorical variables were regarded as a separate group.

Any detailed information about the variables acquisition process is described at www.cdc.gov/nchs/nhanes/.

### Statistical analyses

We used the weighted analysis to obtain national representation following the NCHS Analysis Recommendation. The continuous variables were characterized by mean ± standard deviation, or median and interquartile range when appropriate. The categorical variables were presented as a percentage(%). The P-value was calculated using a weighted chi-squared test for categorical variables and a linear regression model for continuous variables. The association between birth weight and blood pressure, including SBP and DBP, was analyzed using multivariable linear regression. To further analyze the relationship between birth weight and SBP as well as DBP, we used the following three models: Model 1: No adjustment for variables; Model 2: Gender, age, and race/ethnicity were adjusted; Model 3: All underlying covariates including sex, age, race/ethnicity, ratio of family income to poverty, height, weight, BMI, waist circumference, TG, TC, LDL, FBG, UA, Cr, BUN, glycohemoglobin and mothers' age when born were adjusted.

In addition to further data research, we subsequently performed subgroup analysis stratified by age, gender, and race/ethnicity. A weighted generalized additive model and a smooth curve fitting were conducted to address nonlinearity afterward. When nonlinearity was uncovered, we first calculated the vital inflection point using a recursive algorithm and then conducted a weighted two-piecewise linear regression model on both sides of the inflection point. The statistical analyses were performed using R software (version 3.4.3, http://www.Rproject.org) and EmpowerStats software (http://www.empowerstats.com). The results would be considered to be statistically significant if a two-sided p-value of < 0.05.

## Results

Weighted characteristics, including socio-demographic and laboratory data of 7600 responders in the present study, are reported and summarized in Table 1. Among all the research objects, the average was 11.54 ± 2.31 years old. Overall, the proportion of males was slightly higher than females: 51.06% were boys, and 48.94% were girls. A higher proportion non-Hispanic White subjects were included in the study(54.67%) compared with non-Hispanic Black(13.90%) and Mexican Americans (14.90%), and other race/ethnicity(16.54%). In comparison to those with a birth weight less than 88.2 oz, the SBP was much lower in those with heavier birth weights, while DBP was equal in the two groups. Pulse, height, weight, mother's age when born, and the ratio of family income to poverty were all of great statistical significance between the two groups. In addition, those with a birth weight more than 88.2 oz tend to have a high proportion of obesity in their later life when compared to those with a birth weight less than 88.2 oz(24.60% vs. 18.50%). Detailed descriptions of the participants in the study are listed in Table 1.

**Table 1:** Detailed descriptions of the participants included in the present study

Birth weight	< 88.2 *N* = 732	> = 88.2 *N* = 6868	All *N* = 7600	*p* value
Age(years old)	11.58 ± 2.34	11.54 ± 2.30	11.54 ± 2.31	0.6823
Birth weight(ounces)	72.20 ± 15.42	121.18 ± 16.93	117.07 ± 21.62	< 0.0001
mothers' age when born (years old)	26.64 ± 6.68	27.32 ± 6.01	27.26 ± 6.07	0.0066
pulse(bpm)	79.41 ± 11.76	80.75 ± 12.10	80.64 ± 12.08	0.0074
SBP(mmHg)	105.06 ± 10.12	104.09 ± 9.40	104.17 ± 9.47	0.0132
DBP(mmHg)	54.70 ± 14.33	54.12 ± 15.10	54.17 ± 15.04	0.3520
Weight(kg)	48.54 ± 19.38	50.48 ± 18.94	50.32 ± 18.99	0.0138
Height(cm)	150.05 ± 14.73	152.21 ± 14.60	152.03 ± 14.62	0.0004
Waist circumference(cm)	72.70 ± 15.34	74.00 ± 14.26	73.89 ± 14.36	0.0308
BMI group(%)				0.034
Normal range	65.03	59.11	59.64	
Overweight	16.47	16.29	16.30	
Obesity	18.50	24.60	24.06	
TG(mmol/L)	0.84 ± 0.24	0.84 ± 0.25	0.84 ± 0.25	0.9404
LDL_C(mmol/L)	2.21 ± 0.29	2.21 ± 0.30	2.21 ± 0.30	0.9296
TC(mmol/L)	4.10 ± 0.65	4.06 ± 0.65	4.06 ± 0.65	0.0854
HDL_C(mmol/L)	1.40 ± 0.34	1.38 ± 0.32	1.38 ± 0.32	0.1074
Uric acid(umol/L)	291.09 ± 68.56	291.81 ± 71.30	291.74 ± 71.03	0.529
Creatinine(umol/L)	58.02 ± 12.85	57.54 ± 12.67	57.59 ± 12.69	0.911
Blood urea nitrogen(mmol/L)	3.83 ± 1.30	3.77 ± 1.16	3.78 ± 1.17	0.698
glycohemoglobin(%)	5.29 ± 0.23	5.27 ± 0.26	5.28 ± 0.26	0.1178
Ratio of family income to poverty	2.24 ± 1.50	2.52 ± 1.57	2.50 ± 1.57	< 0.0001
Gender(%)				0.0519
Male	47.38	51.40	51.06	
Female	52.62	48.60	48.94	
Race/ethnicity (%)				< 0.0001
Mexican American	14.92	14.89	14.90	
Other race	16.30	16.56	16.54	
Non-Hispanic Whites	45.68	55.49	54.67	
Non-Hispanic Blacks	23.10	13.06	13.90	

**Fig. 1:**
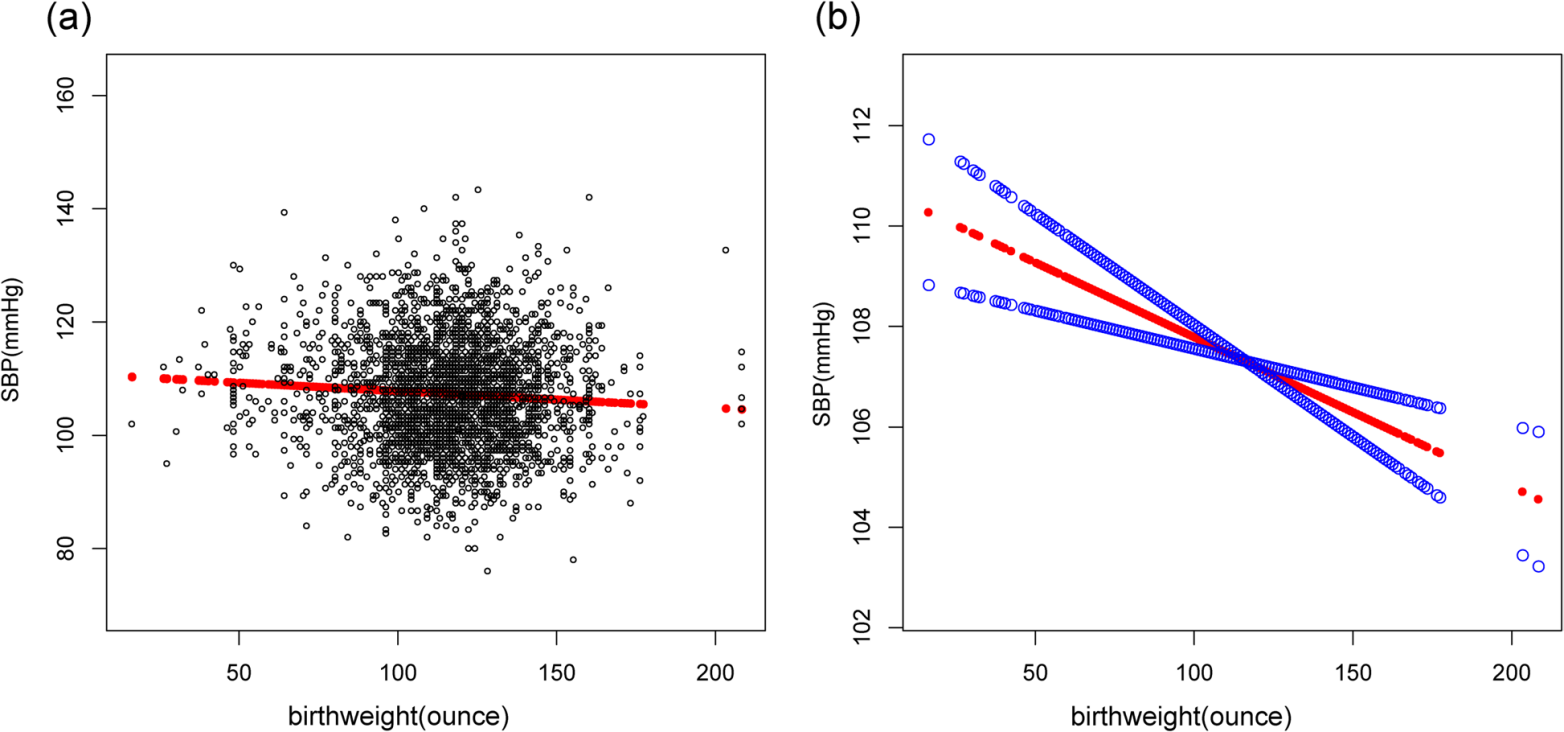
Relationship between birth weight and SBP. **a** Each black point represents a sample. **b** The red line represents the smooth curve fit between variables. In comparison, blue bands represent the 95% CI. Sex, age, race/ethnicity, ratio of family income to poverty, height, weight, BMI, waist circumference, TG, TC, LDL, FBG,UA, Cr, BUN, glycohemoglobin and mothers' age when born were adjusted

A clear inverse relationship can be observed between birth weight and SBP in the fully-adjusted model(β = -0.02, 95%CI: -0.04 to -0.04, *p* = 0.0013). In sub-analysis stratified by gender, age, race/ethnicity, this negative association was more obvious in males(β = -0.03, 95%CI: -0.05 to -0.01, *p* = 0.0027), and age >  = 13 years old(β = -0.03, 95%CI: -0.04 to -0.01, *p* = 0.0027) and in non-Hispanic White (β = -0.03, 95%CI: -0.06 to -0.00,*p* = 0.0446) (Table2, Fig. 1).

**Table 2:** Association between birth weight (ounces) and SBP (mmHg)

Outcome	Model 1, β(95% CI), *p*	Model 2, β(95% CI), *p*	Model 3, β(95% CI), *p*
SBP	-0.01 (-0.02, 0.00) 0.0828	-0.01 (-0.02, -0.00) 0.0190	-0.02 (-0.04, -0.01) 0.0013
quartiles of birthweight			
Lowest quartile	Reference	Reference	Reference
Q2	-0.22 (-0.80, 0.36) 0.4543	-0.48 (-1.39, 0.43) 0.2992	-0.48 (-1.39, 0.43) 0.2992
Q3	-0.10 (-0.73, 0.53) 0.7618	-0.08 (-0.67, 0.52) 0.8023	-0.64 (-1.57, 0.28) 0.1706
Q4	-0.40 (-1.01, 0.20) 0.1915	-0.52 (-1.10, 0.05) 0.0749	-1.58 (-2.47, -0.69) 0.0005
*p* for trend	0.313	0.115	< 0.001
Stratified by race			
Mexican American	-0.01 (-0.03, 0.01) 0.5126	-0.01 (-0.03, 0.01) 0.2176	-0.03 (-0.06, 0.00) 0.0591
Other Race/ethnicity	0.00 (-0.02, 0.02) 0.8436	-0.00 (-0.02, 0.02) 0.8918	-0.03 (-0.06, 0.00) 0.0563
Non-Hispanic White	-0.01 (-0.03, 0.01) 0.3019	-0.02 (-0.04, -0.00) 0.0433	-0.03 (-0.06, -0.00) 0.0446
Non-Hispanic Black	-0.00 (-0.02, 0.02) 0.6653	-0.01 (-0.03, 0.01) 0.2486	-0.03 (-0.06, 0.00) 0.0605
Stratified by age			
Age < 13	-0.01 (-0.02, -0.00) 0.0424	-0.01 (-0.02, 0.00) 0.0505	-0.03 (-0.08, 0.02) 0.1954
Age > = 13	-0.01 (-0.02, 0.01) 0.3770	-0.01 (-0.03, 0.01) 0.2467	-0.03 (-0.04, -0.01) 0.0027
Stratified by gender			
male	-0.00 (-0.02, 0.01) 0.5182	-0.00 (-0.02, 0.01) 0.6287	-0.03 (-0.05, -0.01) 0.0027
female	-0.02 (-0.04, -0.01) 0.0015	-0.02 (-0.03, -0.01) 0.0034	-0.02 (-0.06, 0.02) 0.1519

Model 1: No adjustment for variables;

Model 2: Gender, age, and race/ethnicity were adjusted;

Model 3: All underlying covariates including sex, age, race/ethnicity, ratio of family income to poverty, height, weight, BMI, waist circumference, TG, TC, LDL, FBG, UA, Cr, BUN, glycohemoglobin and mothers' age when born were adjusted

**Fig. 2:**
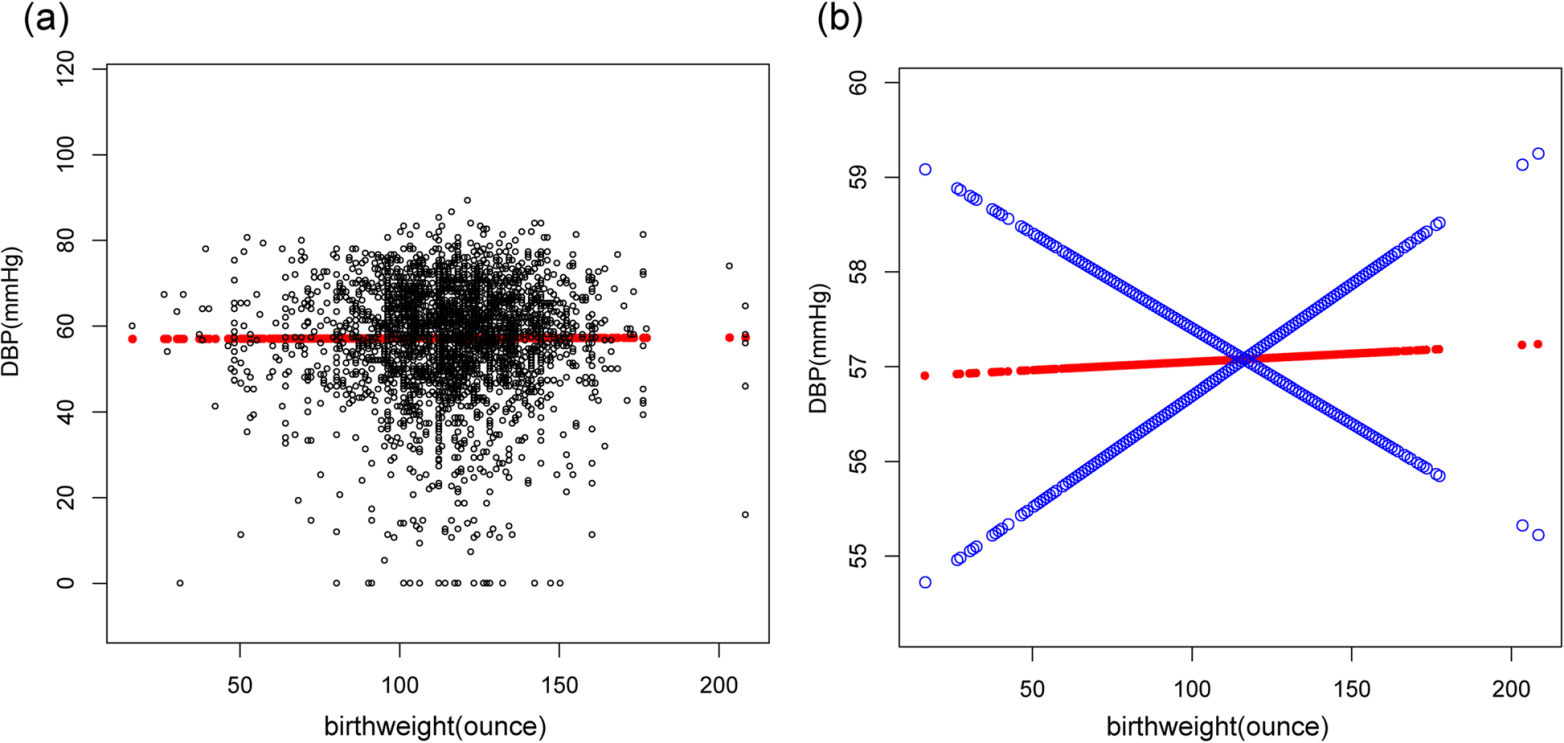
Association between birth weight and DBP. **a** Each black point represents a sample. **b** The red line represents the smooth curve fit between variables. In comparison, blue bands represent the 95% CI. Sex, age, race/ethnicity, ratio of family income to poverty, height, weight, BMI, waist circumference, TG, TC, LDL, FBG,UA, Cr, BUN, glycohemoglobin and mothers' age when born were adjusted

Contrastingly and unexpectedly, there was no linear association between birth weight and DBP in the fully adjusted model(β = -0.01, 95%CI: -0.03 to 0.02, *p* = 0.5668) and sub-analysis stratified by gender, age, race/ethnicity(Table 3, Fig. 2).

**Table 3:** Association between birth weight(ounces) and DBP (mmHg)

Outcome	Model 1, β(95% CI), *p*	Model 2, β(95% CI), *p*	Model 3, β(95% CI), *p*
DBP	0.02 (0.00, 0.03) 0.0316	0.02 (0.00, 0.03) 0.0126	-0.01 (-0.03, 0.02) 0.5668
quartiles of birthweight			
Lowest quartile	Reference	Reference	Reference
Q2	0.30 (-0.68, 1.28) 0.5512	0.38 (-0.56, 1.32) 0.4265	0.16 (-1.21, 1.52) 0.8214
Q3	0.42 (-0.58, 1.42) 0.4098	0.50 (-0.46, 1.46) 0.3071	-0.27 (-1.66, 1.12) 0.7049
Q4	1.30 (0.34, 2.26) 0.0078	1.45 (0.52, 2.38) 0.0022	-0.01 (-1.35, 1.33) 0.9884
*p* for trend	0.008	0.002	0.851
Stratified by race			
Mexican American	0.01 (-0.02, 0.04) 0.4077	0.01 (-0.02, 0.04) 0.3730	-0.01 (-0.05, 0.03) 0.7140
Other Race/ethnicity	0.01 (-0.03, 0.04) 0.7122	0.01 (-0.02, 0.04) 0.3905	-0.01 (-0.05, 0.03) 0.6697
Non-Hispanic White	0.02 (-0.01, 0.05) 0.2214	0.02 (-0.01, 0.05) 0.2025	-0.01 (-0.05, 0.04) 0.7183
Non-Hispanic Black	0.03 (-0.00, 0.06) 0.0969	0.03 (-0.00, 0.06) 0.0566	0.02 (-0.02, 0.07) 0.3411
Stratified by age			
AGE group = < 13	0.01 (-0.01, 0.03) 0.4244	0.02 (0.00, 0.04) 0.0278	0.01 (-0.04, 0.06) 0.7507
AGE group = > = 13	0.01 (-0.00, 0.03) 0.0530	0.01 (-0.01, 0.04) 0.2306	-0.00 (-0.03, 0.02) 0.8407
Stratified by gender			
male	0.02 (-0.00, 0.04) 0.0874	0.02 (-0.00, 0.04) 0.0515	-0.00 (-0.03, 0.03) 0.8908
female	0.02 (0.01, 0.04) 0.0051	0.02 (-0.00, 0.04) 0.1179	0.00 (-0.03, 0.03) 0.8449

Model 1: No adjustment for variables;

Model 2: Gender, age, and race/ethnicity were adjusted;

Model 3: All underlying covariates including sex, age, race/ethnicity, ratio of family income to poverty, height, weight, BMI, waist circumference, TG, TC, LDL, FBG, UA, Cr, BUN, glycohemoglobin and mothers' age when born were adjusted

**Fig. 3:**
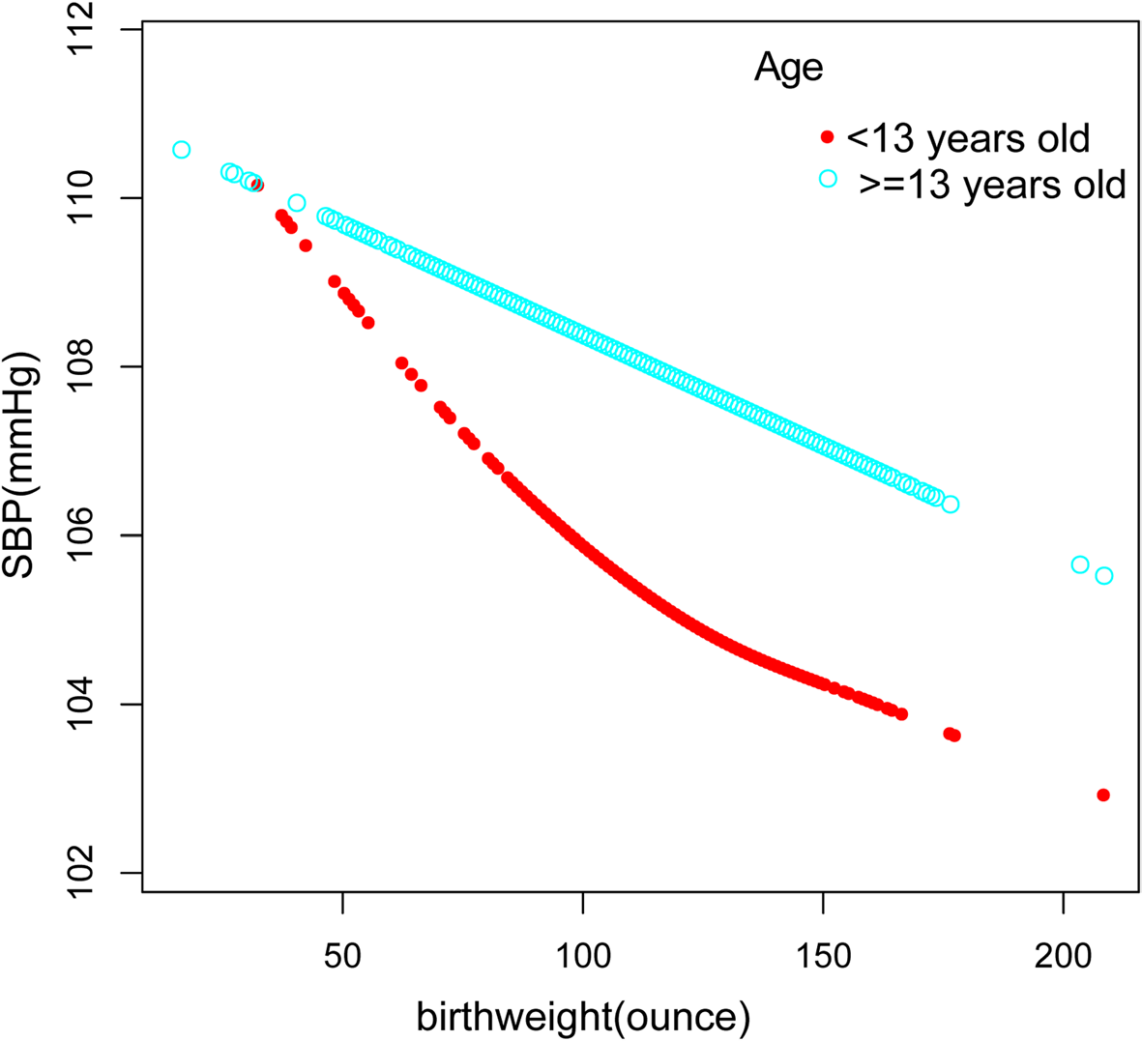
The relationship between birth weight and SBP, stratified by age

**Fig. 4:**
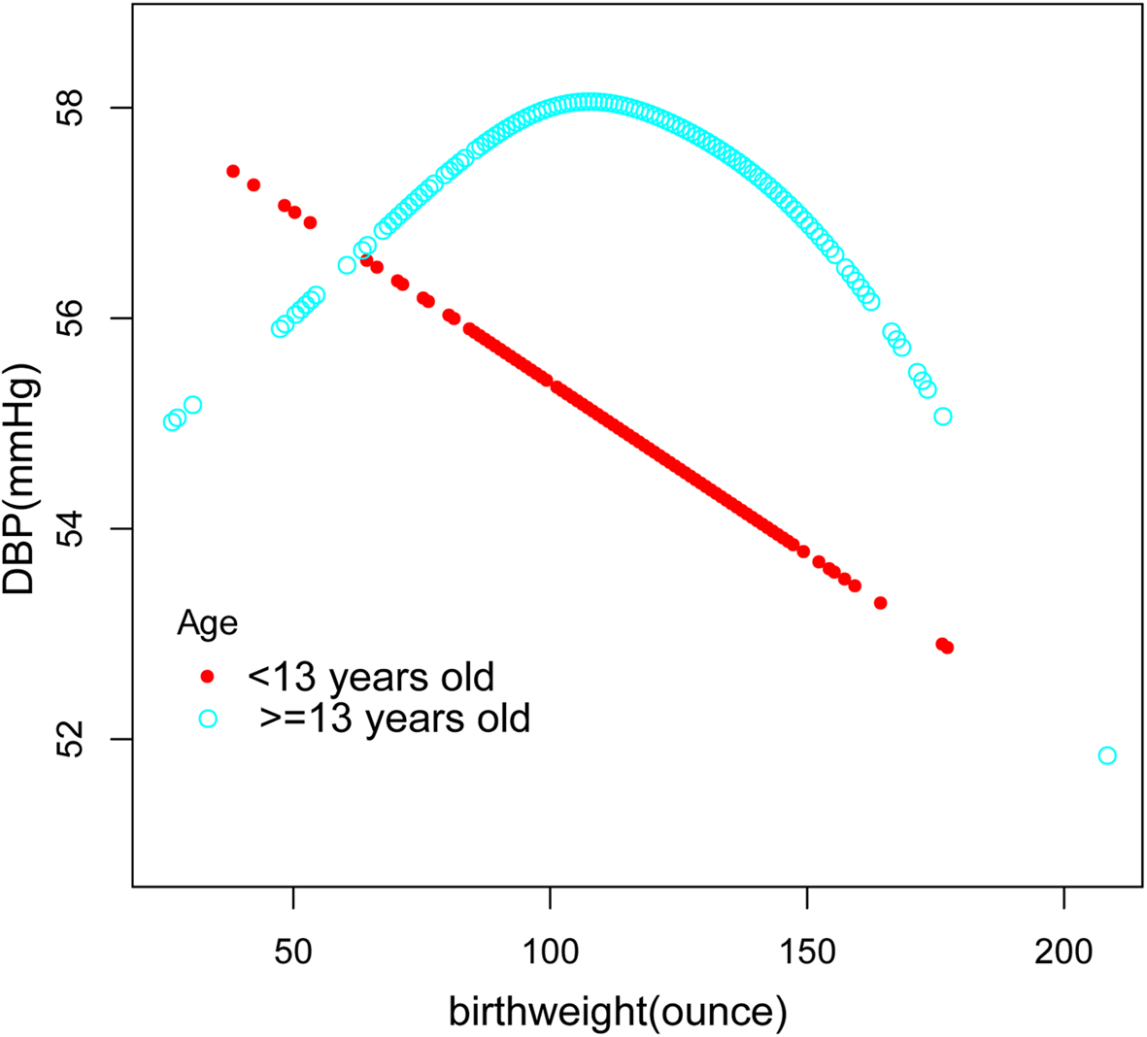
The association between birth weight C and DBP, stratified by age

**Fig. 5:**
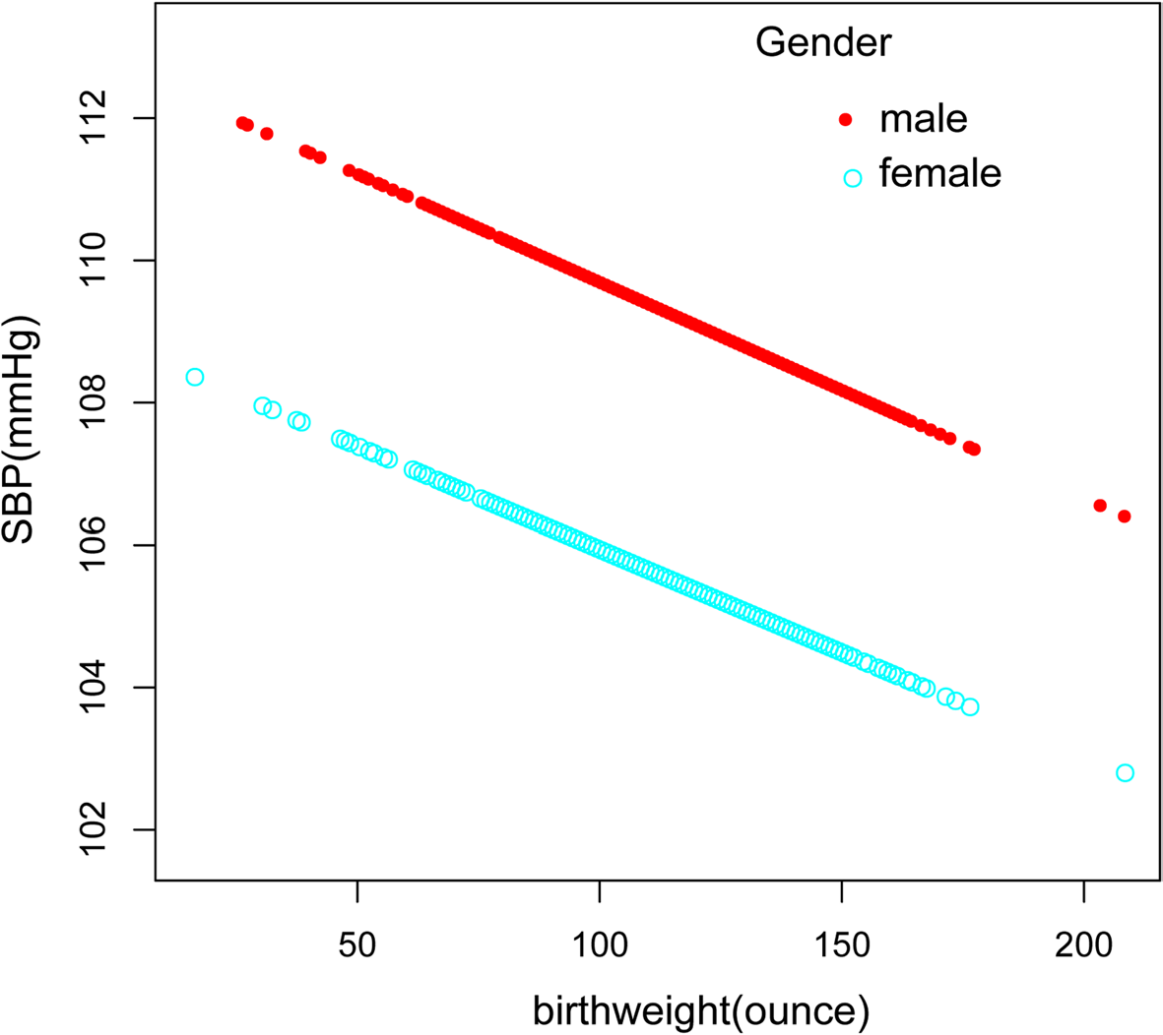
The relationship between birth weight and SBP, stratified by gender

**Fig. 6:**
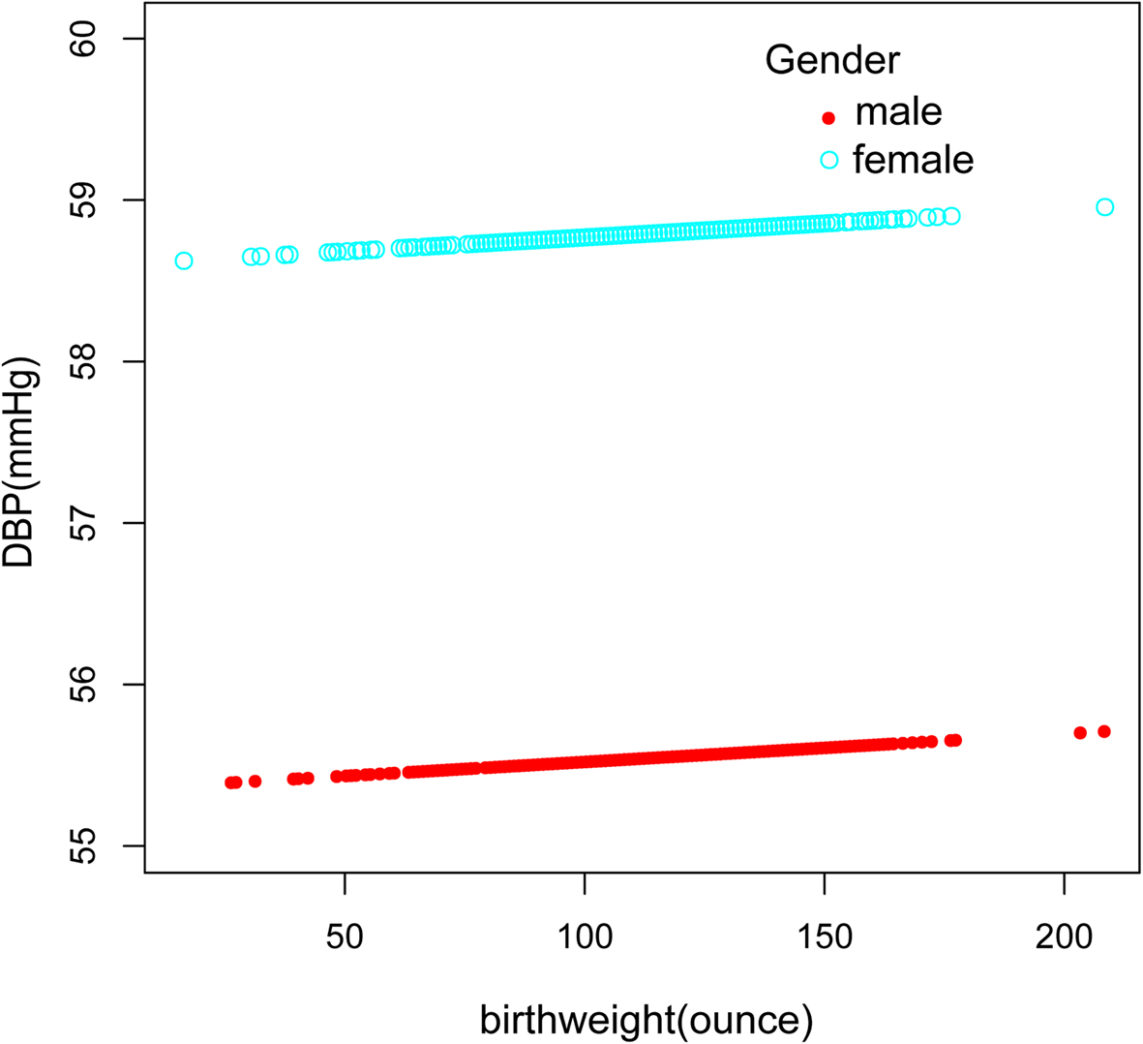
The association between birth weight C and DBP, stratified by gender

**Fig. 7:**
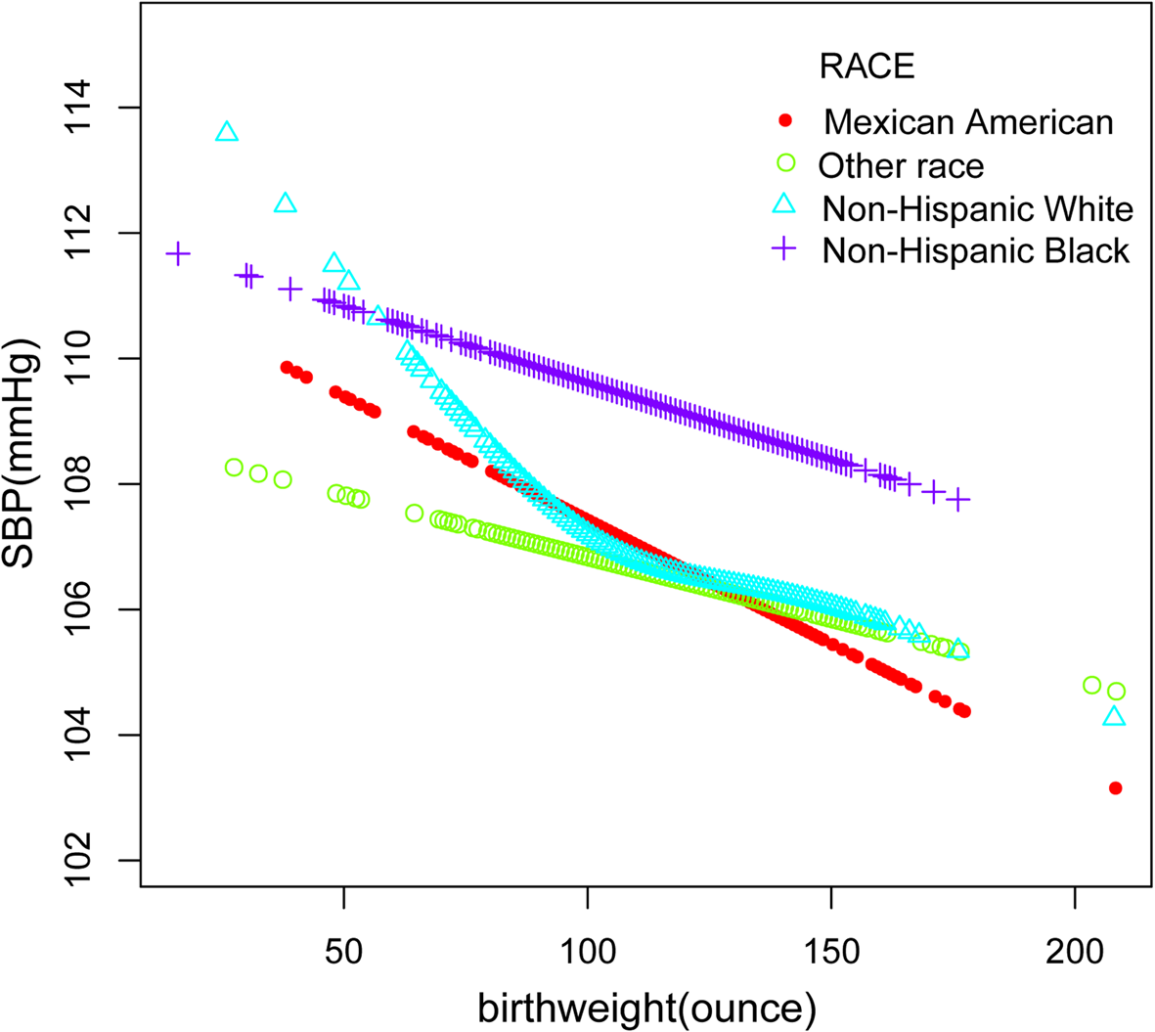
The relationship between birth weight and SBP, stratified by race/ethnicity

**Fig. 8:**
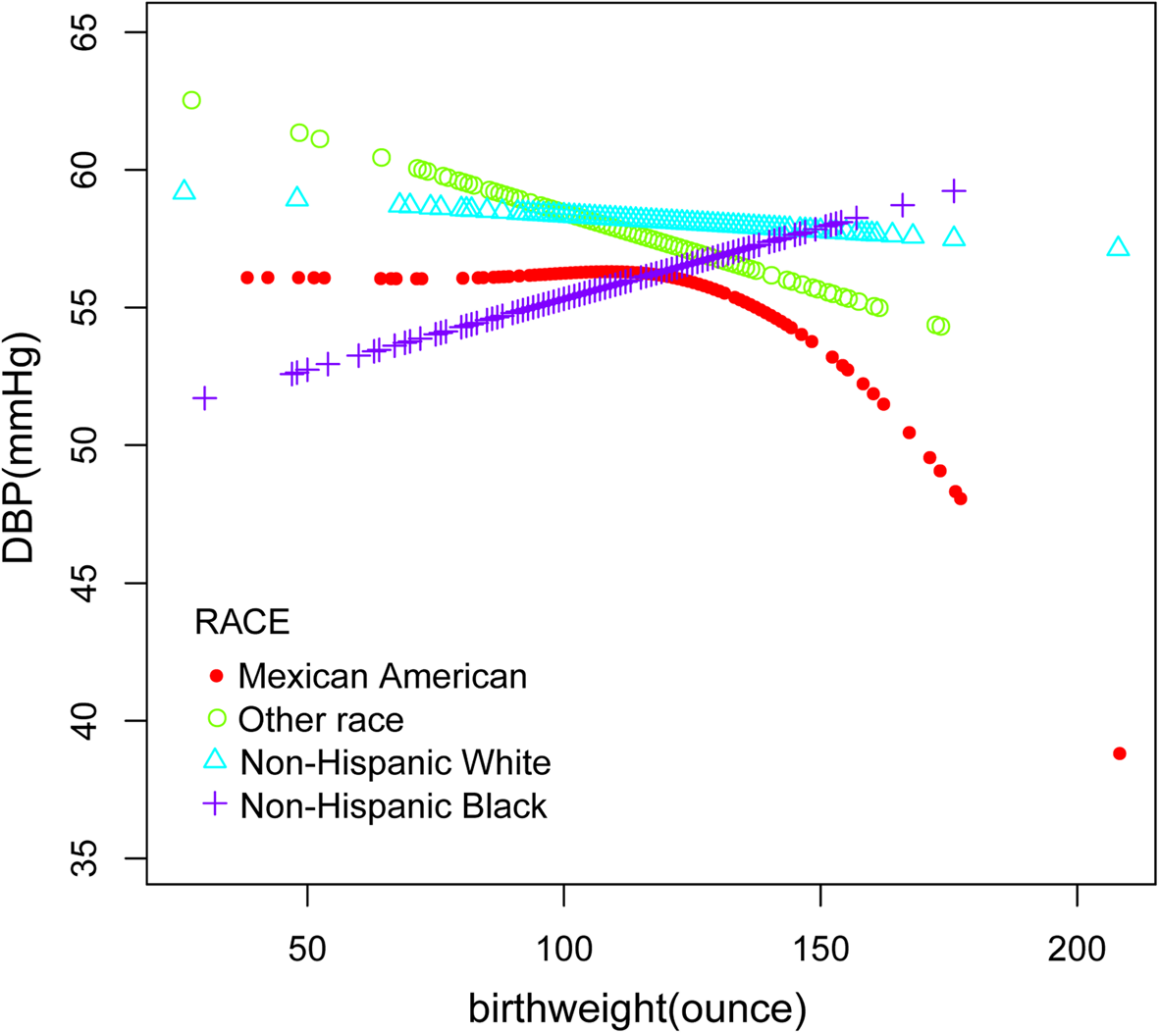
The association between birth weight C and DBP, stratified by race/ethnicity

Furthermore, we performed a weighted generalized additive model and a smooth curve fitting stratified by age, gender, and race/ethnicity to detect the nonlinear relation between birth weight and SBP as well as DBP and further confirm the results (Fig. 3,4,5,6,7,8). An inverted U-shaped and J-shaped relationship was uncovered between birth weight and DBP in those aged 13 or above and Mexican Americans, respectively (Fig. 4, Fig. 8). According to the smoothing plot, we further performed a two-piecewise linear regression model to address birth weight's threshold effect on DBP. The inflection point calculated by a recursive algorithm of birth weight in these groups was all 105 oz (Table 4).

**Table 4:** Threshold effect analysis of birth weight and DBP using two-precise linear regression

DBP	Adjustedβ(95% CI), *p*
**age > = 13 years**	
Fitting by a standard linear model	-0.00 (-0.03, 0.02) 0.7811
Fitting by two precise linear model	
Inflection point	105
birthweight < 105 oz	0.01 (-0.04, 0.06) 0.7194
birthweight > 105 oz	-0.01 (-0.05, -0.03) 0.0322
Log-likelihood ratio	0.583
**Mexican American**	
Fitting by a standard linear model	-0.01 (-0.06, 0.03) 0.5586
Fitting by two precise linear model	
Inflection point	105
birthweight < 105 oz	0.01 (-0.28, 0.19) 0.8437
birthweight > 105 oz	-0.13 (-0.51, -0.07) 0.0258
Log-likelihood ratio	0.026

Sex, age, race/ethnicity, ratio of family income to poverty, height, weight, BMI, waist circumference, TG, TC, LDL, FBG, UA, Cr, BUN, glycohemoglobin and mothers' age when born were adjusted

## Discussion

The present study utilized the population-based cross-sectional resources of NHANES to detect the correlation between birth weight and blood pressure in general children and adolescents aged 8 to 15, which, to our knowledge, was the first time to examine such a relationship in this population. We adjusted the socioeconomic conditions, physical measurement indicators, mother's age when born, and common predisposing risk factors for hypertension for all the participants. The major finding to emerge from the current study was that birth weight was negatively correlated with SBP even after adjusting for the relevant covariates, especially in White, aged between 13 to 15, and male individuals. However, there was no unidirectional relationship between birth weight and DBP. A somewhat unexpected finding is that for individuals aged between 13 to 15 and Mexican Americans, birth weight and DBP showed an inverted U-shaped relationship and an inverted J-shaped relationship, respectively.

Globally, hypertension, responsible for the increasing burden of disease, is one of the main threats to public health and a significant risk factor that predisposes individuals to various cardiovascular conditions [1, 24]. Hypertension in the young is particularly complex and challenging because it is demonstrated an increased risk of developing cardiovascular disorders later in adulthood [8, 25, 26]. As we all know, hypertension is a multifactorial disorder involving the combined effect of environment and genes with an unsatisfactory controlling rate [10, 11]. Continuing to explore the etiology of hypertension, especially in adolescence and childhood, has significant implications for preventing disease progression and improving global health. Low body weight, an indicator of congenital development dysplasia or an adverse intrauterine environment, has recently been thought to be widely implicated in high blood pressure and other cardiovascular diseases [27, 28].

Barker et al. [29] first proposed the hypothesis of birth weight and later cardiovascular risk in adulthood. A large number of subsequent studies also confirmed the conclusions of the predecessors [30–33]. A Mendelian randomized study aimed at elucidating complex underlying biological mechanisms further corroborated previously published work [30]. The study identified a causal relationship between low birth weight and susceptibility to cardiovascular disease and diabetes, independent of adult obesity or high blood pressure. Recently, studies have further indicated that birth weight had a negative relationship with blood pressure, and low birth weight might lead to the risk of late increased blood pressure in adulthood [12–14, 28, 34, 35]. In agreement with earlier findings, the negative correlation between birth weight and SBP was also uncovered in children and adolescents even after adjusting for other relevant confounders, including the mother's age when born that may possess an elevation in blood pressure in the present study.

The following underlying mechanism may be involved in the reverse relationship between birth weight and blood pressure. Firstly, low birth weight caused by fetal dysplasia is a significant cause of increased blood pressure in later life. Low birth weight is considered an essential surrogate indicator of a poor and adverse intrauterine environment [36, 37]. Later, scholars advanced this theory to integrate the critical role of the kidney, indicating that the decrease in the number of nephrons at birth was a significant cause for diseases such as hypertension and coronary heart disease in adolescents and adulthood [12, 16, 38, 39]. In 2003, the kidney collected by Hughson M et al. [40] through autopsy confirmed that people with low birth weight had fewer nephrons, providing further evidence for the reverse association between birth weight and blood pressure. In addition, the developmental origin theory may further explain the negative relationship between birth weight and blood pressure, which believed that cardiovascular diseases originate from intrauterine development, not just traditional acquired [41].

Nevertheless, some studies have questioned the relationship between birth weight and blood pressure, and some scholars even draw contradictory conclusions [15–17, 42]. Inadequate adjustment of related variables and nonrepresentative study samples are the main reasons that may contribute to discrepant results [43]. Our research object is the nationally representative NHANES, and after adjusting BMI, weight, height, waist circumference, and common risk factors for high blood pressure such as FBG and blood lipids, this negative correlation between birth weight and SBP still exists. Moreover, we performed a subgroup analysis of different groups, and the same conclusions could be drawn.

Previous findings have revealed that birth weight was identified to be associated inversely with DBP in adults [12] and adolescents [44]. This negative correlation, conversely, has not been replicated in children and adolescents in the current research. Our findings suggested a nonidentical relationship between birth weight and DBP, which may imply that the influence of birth weight on DBP may be the result of a combination of multiple factors, including growth restriction in utero and acquired factors. Similar to our findings, an analysis of data from a census in six major urban areas in China showed that birth weight did not influence DBP [16]. Since China is a predominantly agricultural country, the objects of this study mainly collected from urban areas without rural regions, so the sample may not be nationally representative. The NHANES data used in our research is a typical nationally representative database based on demographic characteristics. We found this nonlinear relationship between birth weight and DBP and that the effect persisted after adjusting for potential confounding factors. Unlike SBP, DBP is not affected by birth weight, highlighting different potential mechanisms behind high SBP and high DBP in the young [16, 45]. As a result, more animal models and clinical studies are required to explain this relationship between birth weight and DBP in our observations.

In addition, we found that among participants at the age of 13 to 15 years and Mexican Americans, there was an inverted U-shaped and inverted J-shaped relationship between birth weight and blood pressure. Furthermore, these trends were not observed in those younger than 13, which may be related to different physical growth manner after the age of 13, such as changes in adolescent hormones, which requires further confirmation. Previous studies have found that different groups of people had different responses to blood pressure. For example, Keller G et al. [46] analyzed 10 patients with essential hypertension who died unexpectedly and found that White had significantly fewer nephrons. Our cross-sectional study found that birth weight and DBP formed a reverse J-shaped relationship in Mexican Americans. Specifically, when the birth weight was less than 105 oz, the effect on DBP was little. While the birth weight was greater than 105 oz, the diastolic blood pressure decreased by 1.06 mmHg for every ounce increase in birth weight. Additionally, our study also implies that for those aged 13 to 15, a birth weight less than 105 oz may contribute to an elevation in blood pressure.

To conclude, the current study identified that birth weight was negatively related to SBP but not significantly related to DBP in children and adolescents aged 8 to 15 during the NHANES 2007–2018. However, we observed an inverted U-shaped association between birth weight and DBP, with low and high levels being related to lower DBP in individuals aged 13 to 15. In addition, our current study indicates a reverse J characteristic curve between birth weight and DBP in Mexican Americans. It is well known that even a slight rise in blood pressure can increase the long-term risk of target organ damage and cardiovascular disease. Therefore, it is imperative to tease out the etiology of hypertension in the young and find potential preventive measures. Our findings have a number of profound implications in clinical practice. In the first place, our research supports the view that using precision medical measures to improve prenatal nutrition and growth to intervene in low-weight fetuses may improve cardiovascular fitness in adulthood. Secondly, for infants with low birth weight, standardized monitoring of blood pressure and timely intervention, such as nutrition and diet adjustments, may help reduce the risk of hypertension, exceptionally high SBP. Lastly, birth weight has different effects on DBP for different groups of people, suggesting that targeted intervention measures should be taken rather than generalizations.

Nevertheless, this study still has the following primary deficiencies. First, this is a cross-sectional study, and the birth weight data reported by the agency may have some recall bias. Second, there is a lack of dynamic information regarding the impact of birth weight on adolescents' blood pressure. Finally, the absence of data about underlying confounding factors such as gestational age, puberty development, physical activities, and other unidentified prognostic factors was another flaw of the study. Our findings should be interpreted with caution, given the above limitations. Prospective studies examining infants and tracking their blood pressure in adulthood to verify the causal effect of birth weight on hypertension are imperative and urgent.

## Data Availability

The data used to support the findings of this study are available from corresponding author upon request.
